# Long term development of diastolic dysfunction and heart failure with preserved left ventricular ejection fraction in heart transplant recipients

**DOI:** 10.1038/s41598-022-07888-9

**Published:** 2022-03-09

**Authors:** Pimprapa Vejpongsa, Guillermo Torre-Amione, Hernan G. Marcos-Abdala, Salil Kumar, Keith Youker, Arvind Bhimaraj, Sherif F. Nagueh

**Affiliations:** Houston Methodist Hospital, 6550 Fannin, SM-1832, Houston, TX 77025 USA

**Keywords:** Cardiology, Signs and symptoms

## Abstract

Heart transplant recipients (HTX) have several risk factors for heart failure which can trigger pro-inflammatory and fibrosis factors and set into motion pathophysiologic changes leading to diastolic dysfunction and HFpEF. The objective of the study was to determine if HTX recipients with dyspnea have diastolic dysfunction and HFpEF. Twenty-five HTX were included. LV systolic and diastolic functions were evaluated using conductance catheters to obtain pressure volume loops. LV function was assessed at rest and during moderate intensity exercise of the upper extremities. A significant increase occurred in LV minimal pressure (3.7 ± 3.3 to 6.5 ± 3.5 mmHg) and end diastolic pressure or EDP (11.5 ± 4 to 18 ± 3.8 mmHg, both *P* < 0.01) with exercise. With exercise, the time constant of LV relaxation shortened in 2, was unchanged in 3, and increased in the remaining patients (group results: rest 40 ± 11.6 vs 46 ± 9 ms, *P* < 0.01). LV chamber stiffness constant was abnormally increased in all but 2 patients. Indices of LV systolic properties were normal at rest but failed to augment with exercise. In 15 who agreed to blood draw, inflammation and fibrosis markers were obtained. A significant association was observed between LV EDP and Pro-Col III N-terminal (r = 0.58, *P* = 0.024) and IL-1-soluble receptor (r = 0.59, *P* = 0.02) levels. HTX have diastolic dysfunction and can develop HFpEF several years after cardiac transplantation. The abnormally increased LV chamber stiffness and the prolongation or lack of shortening of the time constant of LV relaxation with exercise are the underlying reasons behind the observed changes in LV diastolic pressures with exercise.

## Introduction

Heart transplant (HTX) recipients have limited exercise tolerance years after transplantation, despite normal left ventricular (LV) ejection fraction (EF) and normal LV filling pressures at rest. There are few studies early after transplantation including few patients, reporting abnormal LV diastolic pressures^1,2^, and deficient acceleration of relaxation with exercise^3^. Data on long term results are more limited being based on mitral inflow by Doppler echocardiography which has major limitations as an index of left ventricular (LV) diastolic function in HTX recipients^4^. We hypothesized that HTX recipients can develop diastolic dysfunction and HFpEF. We thus sought to prospectively assess LV systolic and diastolic properties using the invasive gold standard of pressure volume loops in HTX.

## Methods

### Patient population

All HTX recipients ≥ 5 years after surgery (all patients included were transplanted between 7/5/2001 and 1/26/2015) undergoing routine surveillance catheterization were prospectively evaluated over the past 18 months as per the standard practice at our institution. Patients with heparin allergy (in whom heparinized saline cannot be used during left heart catheterization or LHC), history of percutaneous coronary intervention or myocardial infarction after transplantation, significant coronary artery disease at the time of LHC, active bleeding problems, any graft dysfunction defined by LV EF < 50% since transplantation, valvular heart disease (≥ moderate regurgitation), non-sinus rhythm, ESRD on hemodialysis (as arterio-venous communication can cause high output heart failure), and inability or refusal to give informed consent were excluded. None of the patients had mitral or aortic stenosis. Excluded were 5 patients who refused to consent, one with heparin allergy, 5 on hemodialysis, and 5 with CAD. While including patients with CAD and those with previous PCI or graft dysfunction could have resulted in a more representative sample, these are patients who a priori are known to have myocardial disease. The study was approved by The Methodist Hospital Research Institute IRB, and all patients provided informed consent. All methods were performed in accordance with the relevant guidelines and regulations.

### Cardiac catheterization and pressure volume loop analysis

**Figure 1:**
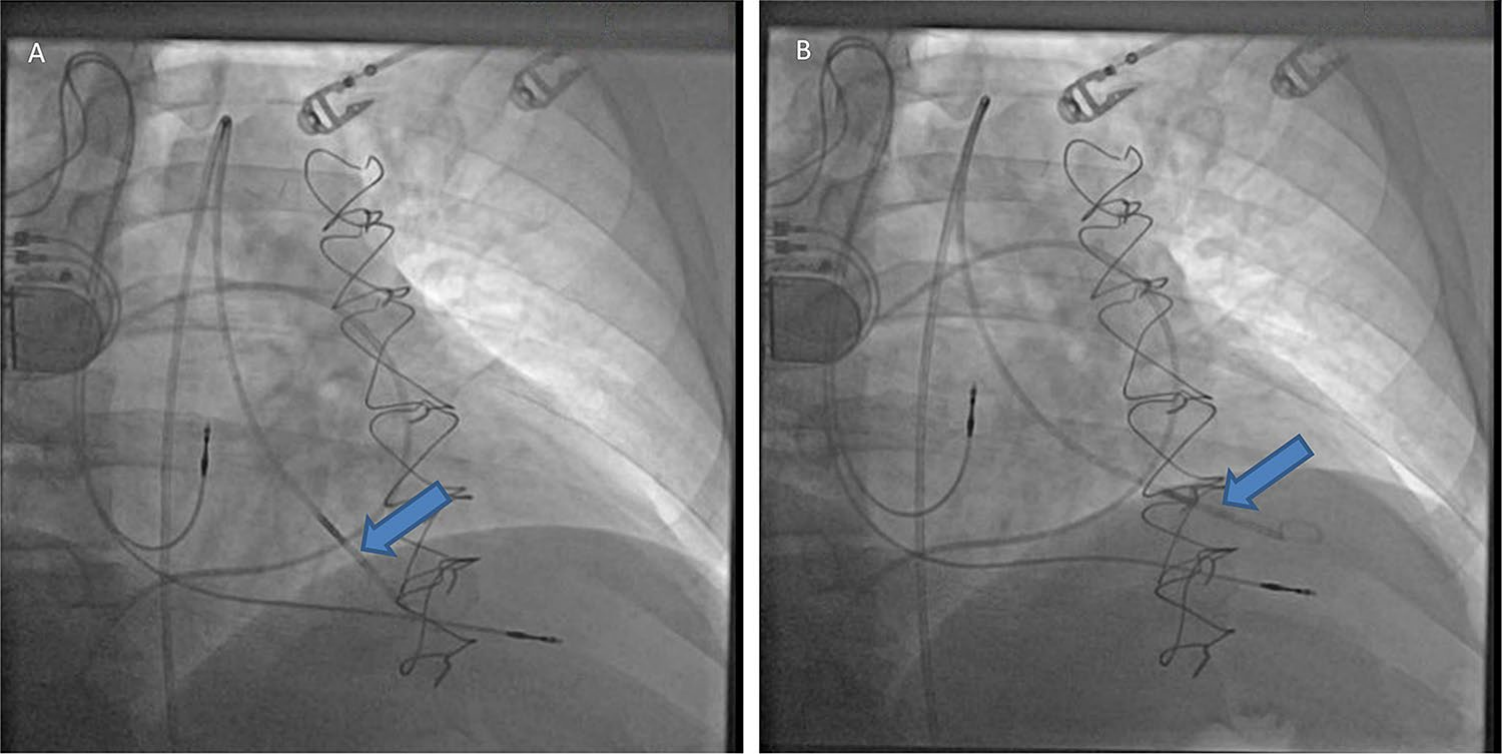
Representative fluoroscopy images showing the 7-French conductance catheter (Blue arrow): (**A**) Prior to alignment with the long axis of the left ventricle and (**B**) After alignment with the long axis of the left ventricle, and with catheter tip placed in left ventricular apex. Patient has dual chamber pacemaker with right atrial and right ventricular leads in the corresponding chambers. The pulmonary artery catheter is already advanced into the pulmonary artery.

Access for LHC was obtained from the femoral artery. After calibration, a 7 French 12-electrode conductance catheter (CD Leycom, Zoetermeer, the Netherlands) was advanced into the LV by crossing the aortic valve using a stiff 0.18 platinum plus™ wire. The catheter was connected to a dedicated system (Inca®, CD Leycom) that continuously acquired and displayed pressure, volume, first derivative of LV pressure versus time, and ECG signals. Proper positioning of the conductance catheter was guided by fluoroscopy, echocardiography, and confirmed by the segmental volume signals. The catheter was placed along the long axis of the LV with its tip in the apex (Fig. 1). Conductance signals were calibrated based on cardiac output measurements using a pulmonary artery (PA) catheter and on determining parallel conductance. Using fluoroscopic guidance, a PA catheter (Swan Ganz) was advanced from the femoral vein into the pulmonary circulation. In addition to measuring right atrial, pulmonary artery pressures, and mean wedge pressure, the PA catheter was used to measure cardiac output (CO) by Fick method, and to determine parallel conductance effect with 5% hypertonic saline injection (10 mL) to convert the conductance signals to LV volumes. Two calibration recordings were obtained and averaged.

**Figure 2:**
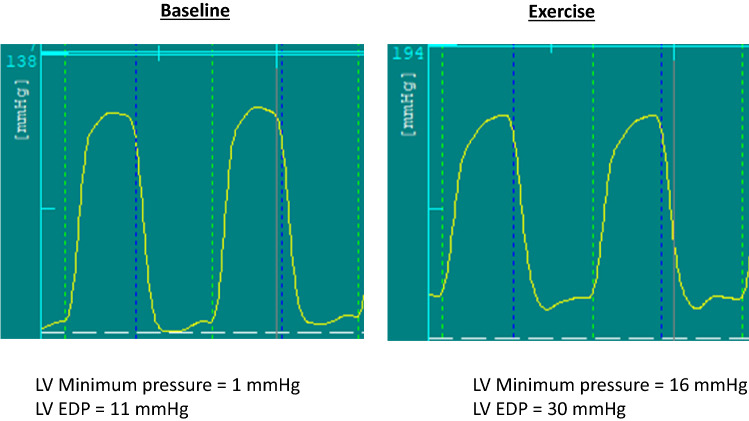
Left ventricular (LV) pressure recordings at baseline shown to the left, and with exercise shown to the right. Notice the marked increase in LV minimum pressure and LV end diastolic pressure (EDP) with exercise. LV peak systolic pressure increased with exercise. Green lines mark end diastole and blue lines mark end systole.

**Figure 3:**
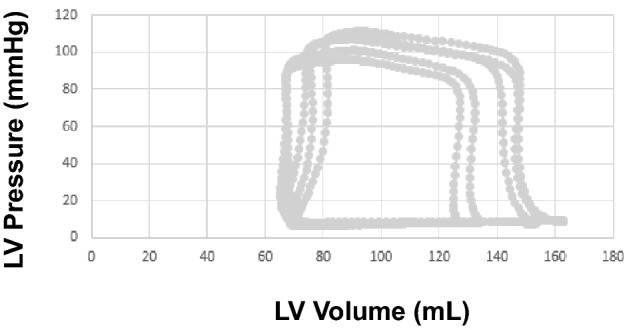
Left ventricular (LV) pressure volume loops during Valsalva from a patient who did not meet HFpEF diagnosis. Stiffness constant (β) was 0.015.

**Figure 4:**
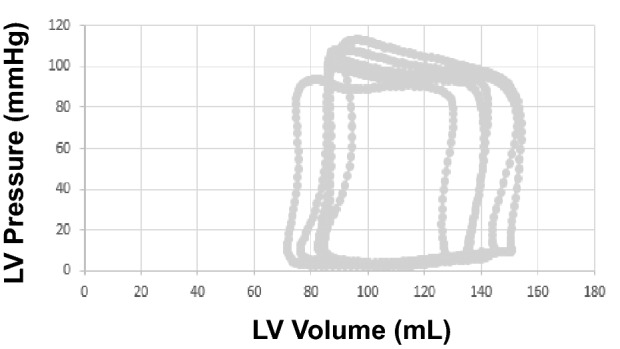
Left ventricular (LV) pressure volume loops during Valsalva. Stiffness constant (β) was increased at 0.0495.

LV pressures and volumes were analyzed at end-expiration. LV minimal, end diastolic (EDP), and systolic pressures were measured (Figs. 2, 3, 4). LV volumes at end-diastole (EDV), end-systole (ESV) and stroke volume (SV) were obtained from the conductance catheter. The time constant of LV relaxation (tau or τ), based on Weiss method^5^ was computed. The correlation coefficient for the curve fits of tau analysis were all ≥ 0.99. LV end systolic pressure/volume relation (ESPVR), preload recruitable stroke work (PRSW), and Starling Contractile Index (SCI) were derived as preload independent indices of LV systolic function. LV passive chamber stiffness (*P* = Ae^βV^) was evaluated based on the stiffness constant (β), where a value exceeding 0.015 indicates abnormally increased chamber stiffness^6^. In addition to single beat derived parameters, patient performed Valsalva maneuver and data obtained during preload decrease were used to obtain pressure volume relationship. This approach has been previously validated against balloon occlusion of inferior vena cava^7^. In addition, stiffness constant was derived by the method of Klotz et al. using single point EDV and EDP^8^. The upper limits of normal for the latter variable is 6.08 based on normal controls without cardiovascular disease and without cardiovascular risk factors^9^. Stroke work from the PV loop along with end systolic pressure and ESPVR was used to obtain PV area (PVA) as a reflection of the total mechanical energy with the expression: PVA = stroke work + 0.5 × (end systolic pressure)^2^/ESPVR^10^.

Moderate intensity exercise (abduction adduction movement of upper limbs while holding 1 kg normal saline bag) of upper extremities (upper extremity exercise was utilized due to lower extremity arterial access) was performed with simultaneous recording of LV pressures and volumes by the conductance catheter until patient exhaustion for duration of 5–10 min^11^. The response to this specific exercise in patients without cardiac dyspnea is no change in mean wedge pressure (normal at baseline at 8 mmHg), along with a significant 26% increase in stroke volume^11^. In comparison patients with HFpEF, develop a significant increase in mean wedge pressure from 11 to 28 mmHg for a similar increase in stroke volume^11^.

### HFpEF definitions

Three definitions of HFpEF based on invasive date were evaluated. These included measurements at rest of LV EDP > 16 mmHg or τ > 48 ms^12^. Definitions using exercise data included end expiratory LV EDP > 23 mmHg, based on upper limits of normal LV EDP with exercise being 23 mmHg, given value of mean EDP with exercise in normal subjects at 13 ± 5 mmHg^13^. The third definition was exercise LVEDP/CO slope > 2 mmHg/Liter per minute^14^. LVEDP was used in place of mean wedge pressure and mean PA pressure since PA pressures were not obtained during exercise. In addition, the noninvasive score H_2_FPEFF score was obtained^15^. This score takes into account age (1 point for age > 60 years), hypertension on ≥ 2 medications (1 point), presence of paroxysmal or persistent atrial fibrillation (3 points), BMI > 30 kg/m^2^ (2 points), pulmonary artery systolic pressure by Doppler echocardiography > 35 mmHg (1 point), and E/e′ ratio by echocardiography > 9 (1 point).

### Echocardiographic imaging and analysis

Patients were imaged simultaneously with ultrasound systems equipped with a multifrequency transducer. Parasternal and apical views were acquired and used to measure LV dimensions, wall thickness, and mass, as well as biplane left atrial (LA) volumes^16^. Duration of diastole was obtained from echocardiographic Doppler recordings as the time interval between aortic valve closure and mitral valve closure. Isovolumetric relaxation time (IVRT), an index of LV relaxation, was measured as the time interval between aortic valve closure and mitral valve opening^17^. In addition, mitral annulus early diastolic velocity (e′) by tissue Doppler (TD), and LA reservoir strain by speckle tracking were measured as indices of diastolic function and LV filling pressures. LV long axis function was assessed using LV global longitudinal strain (GLS) from the three apical views^16^.

### Serum biomarkers

The levels of biomarkers reflecting inflammation, and collagen turnover were measured including: interleukin 1 (IL 1), interleukin 6 (IL 6), tumor necrosis factor alpha (TNF α), C reactive protein (CRP), interleukin 1 receptor type 1 (IL1 R1), growth differentiation factor 15 (GDF-15), procollagen type I N-terminal propeptide (PICP-N-terminal), procollagen type I C-terminal propeptide (PICP-C-terminal), procollagen type III N-terminal propeptide (PIIICP N-terminal), matrix metalloproteinase 1 (MMP 1), and tissue inhibitor of metalloproteinase 1 (TIMP-1). Blood samples were obtained at the time of cardiac catheterization from the patients who consented to drawing blood.

### Statistics

Data are shown as mean ± SD or median with interquartile range (IQR) based on whether they had normal distribution. Categorical data are shown in numbers and percentages. Comparisons between rest and exercise were performed with a 2-tailed paired student t-test or Wilcoxon signed-rank test. Linear regression was used to study relation between biomarkers and diastolic function. Sample size was based on a projected 5 mmHg EDP increment with exercise, SD = 5 mmHg^13^, α = 0.05, and power = 0.9. Statistical significance was defined by *P* < 0.05.

## Results

A total of 25 patients were included in the study with a median of 8 years since cardiac transplantation and more than 3 years since the last rejection episode. The reasons for cardiac transplantation were amyloidosis in 1 patient, peripartum cardiomyopathy in 1 patient, post infarction severe LV dysfunction leading to HFrEF in 7 patients, and non-ischemic cardiomyopathy in the remaining patients. Data acquisition was possible in all patients recruited. Table 1 shows a summary of demographics, clinical data, echocardiographic findings, and right heart catheterization results of the study participants. None had significant epicardial coronary artery disease needing intervention or significant allograft vasculopathy. Patients reported symptoms of fatigue and exertional dyspnea which increased with exercise and led to exercise termination. There were no complications in any of the patients and all were discharged home on the same day as per post cardiac catheterization protocol.

**Table 1:** Summary of clinical, echo, and right heart catheterization findings (N = 25).

Age (years)	60 ± 9
Number of males (%)	21 (84%)
Body mass index (kg/m^2^)	30 ± 4.3
Hypertension (%)	25 (100%)
Diabetes Mellitus (%)	10 (40%)
Hypertension treatment with diuretics	7 (28%)
Hypertension treatment with beta-blockers	4 (16%)
Hypertension treatment with calcium channel blockers	12 (48%)
Hypertension treatment with ACEI or ARB	8 (32%)
Patients on aspirin	24 (96%)
Patients on statins	22 (88%)
≥ 1 episodes of acute cellular rejection grade 2R or 3R	11 (44%)
≥ 1 episodes of acute cellular rejection grade 1R	18 (72%)
Cytomegalovirus status mismatch between donor and recipient hearts	8 (32%)
Patients on Prednisone (dose 5 mg daily)	14 (56%)
Patients on Tacrolimus	21 (84%)
Patients on Rapamycin	2 (8%)
Patients on Cyclosporine	3 (12%)
Patients on Mycophenolate	15 (60%)
Hemoglobin (gm/dl)	14 ± 1.8
Creatinine (mg/dl)	1.2 ± 0.3
Brain natriuretic peptide (pg/ml)	72 ± 36
Average mitral annulus early diastolic velocity (cm/s)	9 ± 2
Average E/e’ ratio	9.8 ± 2.8
Left atrium maximum volume index (mL/m^2^)	43 ± 11
Left atrium reservoir strain (%)	19.6 ± 6.1
Left ventricle global longitudinal strain or GLS (%)	− 16.9 ± 2.6
Mean right atrial pressure (mmHg)	7 ± 3
Pulmonary artery systolic pressure (mmHg)	29 ± 6
Pulmonary artery diastolic pressure (mmHg)	14 ± 5
Mean Pulmonary Artery Pressure (mmHg)	19 ± 5
Mean pulmonary capillary wedge pressure (mmHg)	13 ± 5

*ACEI* Angiotensin converting enzyme inhibitor, *ARB* Angiotensin receptor blocker.

### Echocardiographic findings

LV mass index was normal in all patients, and none had LV hypertrophy, but 22 patients had concentric remodeling (normal LV mass but relative wall thickness > 0.42). LA maximum volume index was increased in all patients (Table 1), which in part is due to remnant of recipient LA. Of note, LA maximum volume index is not a reliable measure of LV diastolic function in the transplant population. LV GLS was within normal values for the group (Table 1).

### Echocardiographic indices of LV diastolic function

No significant relation was present between the change in e’ velocity and the change in the time constant of LV relaxation with exercise. LA reservoir strain was not significantly associated with LV pre-A pressure or LV EDP.

### LV systolic properties at rest and with exercise

All indices of LV systolic properties were within the normal range (Table 2) including LV EF, and the load independent indices of systolic function. Median Vo (volume intercept of LV pressure volume relationship) was 41 mL (25th percentile: 30 and 75th percentile: 63). However, there was no increment in any of the indices with exercise indicating no apparent contractile reserve in this group of patients during the exercise protocol in the catheterization laboratory. Given the flat response of LV systolic function and the increase in LV systolic and arterial pressure with exercise, the ratio of arterial elastance to ESPVR increased with exercise compared to baseline [median 0.7 (25th percentile: 0.5 and 75th percentile: 1) vs median 0.8 (25th percentile: 0.6 and 75th percentile: 1.2), *P* = 0.04]. Expectedly, both stroke work and PVA increased significantly with exercise (Table 1).

**Table 2:** Hemodynamic changes with exercise (N = 25).

	Rest	Exercise
Heart rate(/min)	81 ± 10	86 ± 12*
LV peak systolic pressure (mmHg)	115 ± 14	127 ± 17*
LV end diastolic volume or EDV (mL)	117 ± 25	130 ± 34
LV end systolic volume or ESV (mL)	42 ± 17	51 ± 24*
LV stroke volume (mL)	74 ± 17	76 ± 18
LV ejection Fraction (%)	64 ± 10	61 ± 13
Cardiac output by conductance catheter (L/min)	6 ± 1.2	6.5 ± 1.9
LV minimal pressure (mmHg)	3.7 ± 3.3	6.5 ± 3.5*
LV end diastolic pressure (mmHg)	11.5 ± 4	18 ± 3.8*
Time constant of LV relaxation: τ (ms)	40 ± 11.6	46 ± 9*
LV chamber stiffness constant—single beat	0.03 (0.016–0.05)	0.028 (0.018–0.05)
Klotz stiffness constant (8)	6.2 ± 3.9	6.7 ± 1.2
LV peak filling rate (mL/s)	621 ± 242	640 ± 240
End systolic elastance (E_es_ in mmHg/mL)	2.25 (1.4–3.5)	2 (1.5–3.2)
Preload recruitable stroke Work (mmHg)	53 (43–64)	48.5 (36–66)
Slope of dP/dt versus EDV (mmHg/mL/s)	11 ± 3	10 ± 3.7
Effective arterial elastance (E_a_ in mmHg/mL)	1.3 (1.2–1.6)	1.4 (1.2–2)**
LV arterial coupling (E_a_/E_es_)	0.7 (0.5–1)	0.8 (0.6–1.2)**
External stroke work (mmHg. mL)	7629 ± 2103	8944 ± 2349**
Pressure volume loop area (PVA in mmHg. mL)	9878 (8385–11,501)	12,914 (9701–16,189)*

*P < 0.01; **P < 0.05; *P* > 0.05 for all other comparisons.

### LV diastolic function

Exercise induced a significant increase in LV diastolic pressures (Table 2). τ was > 45 ms in 10 patients (40%) at baseline. With exercise, τ shortened in 2 patients, was unchanged in 3 patients, and increased in 20 patients (increase ≥ 10 ms in 7 patients). A significant correlation was present between τ and IVRT (r = 0.66, *P* < 0.001), but not between τ and duration of diastole. LV chamber stiffness was abnormally increased and exceeded 0.015 in all but 2 patients. Similar findings were noted using multiple beats to obtain β (0.07 ± 0.02). There was no significant change in LV early diastolic peak filling rate with exercise (Table 2).

### Impact of beta-blockers and calcium channel blockers on hemodynamics

The hemodynamic findings at baseline and with exercise were not different between patients who were on beta-blockers/calcium channel blockers and those who were not.

### Relation between biomarkers, and LV diastolic function

In this exploratory analysis, IL-6 was abnormally elevated in most patients. Pro-Col1 N-terminal was abnormally elevated in all, and TIMP 1 in 10 of 15 patients (Table 3). Baseline LV EDP was directly related to Pro-Col III N-terminal (r = 0.58, *P* = 0.024) and IL-1-soluble receptor (r = 0.59, *P* = 0.02) levels.

**Table 3:** Inflammation and fibrosis markers in the study sample (N = 15).

Biomarker	Level	Normal range	Number with abnormally elevated level
1. C reactive protein or CRP	2.9 ± 1.4	< 10 mg/L	0/15
2. IL 1B	5.6 ± 2.9	0.5–12 pg/ml	1/15
3. IL 6	7.4 ± 6.5	0.5–5 pg/ml	9/15
4. IL-1-soluble receptor	6.8 ± 1.7	10–38 pg/ml	0/15
5. TNF-α	6.9 ± 1.2	≤ 8.7 pg/mL	0/15
6. ST2	2.5 ± 0.6	Males: 4–31 ng/ml, Females: 2–21 ng/ml	0/15
7. GDF 15	1.6 ± 0.9	≤ 5 ng/ml	0/15
8. Pro-Col I N-terminal	355 ± 147	100–120 pg/ml	15/15
9. Pro-Col I C-terminal	18 ± 5.3	70 ± 5 ng/ml	0/15
10. Pro-Col III N-terminal	1.7 ± 0.4	2–8 ng/ml	0/15
11. MMP-1	2.4 ± 0.4	2.2–22.9 ng/ml	0/15
12. TIMP1	108 ± 26	58–92 ng/ml	10/15

*IL 1B* Interleukin 1 Beta, *IL 6* Interleukin 6, *IL-1-soluble receptor* Interleukin 1 soluble receptor, *TNF-α* Tumor necrosis factor alpha, *ST2* Interleukin 1 receptor like-1, *GDF-15* Growth differentiation factor-15, *Pro-Col I N-terminal* Procollagen type I N-terminal propeptide, *Pro-Col I C-terminal* Procollagen type I carboxy-terminal propeptide, *Pro-Col III N-terminal* Procollagen type III N-terminal propeptide, *MMP-1* Matrix metalloproteinase 1, *TIMP-1* Tissue inhibitor of metalloproteinase 1.

### Diagnosis of HFpEF

Based on hemodynamic measurements at rest of LV EDP and/or τ^12^, 7 patients (28%) met the diagnostic criteria of HFpEF. Three of the seven patients had LVEDP > 16 mmHg and τ  > 48 ms, whereas 4 patients met the definition based on τ  > 48 ms. All off these 7 patients also had HFpEF based on exercise LVEDP/CO slope > 2 mmHg/Liter per minute^14^. In addition, 10 additional patients met the definition based only on exercise LVEDP/CO slope > 2 mmHg/Liter per minute. Therefore, a total of 17 of the 25 patients (68%) met HFpEF definition based on exercise LVEDP/CO > 2 mmHg/ Liter per minute. Patients who met this definition of heart failure were older, had a higher prevalence of diabetes mellitus, and a longer time since cardiac transplantation but these trends did not reach statistical significance level (*P* > 0.05). The severity and number of rejection episodes, and donor age were not significantly related to hemodynamic measurements. For LV EDP > 23 mmHg with exercise, 8 patients (32%) met the definition of HFpEF.

BNP was < 100 pg/mL in all 7 patients who met HFpEF definition based on hemodynamics measured at rest. It was elevated in only 3 of the 17 patients (18%) with exercise LVEDP/CO slope > 2 mmHg/Liter per minute, and 3 of the 8 patients (38%) with exercise LV EDP > 23 mmHg.

For H_2_FPEF score, the mean was 2 and the median score was 2 (range 0 to 6). There were 3 patients with a score of 3, and 3 patients with score of 4 which denote approximately 50–70% probability of having HFpEF. Only one patient had a score of 6 which denotes a 90% probability of having HFpEF. The remaining patients had 0–2 score indicating low probability for HFpEF.

## Discussion

The normal response to exercise is τ shortening and increase in LV filling rate to maintain adequate LV filling and to increase exercise stroke volume, as diastole duration is shortened with exercise. Importantly, LA pressure does not exceed the upper limits of normal rest pressure in healthy subjects, except at very high cardiac output, allowing normal subjects to exercise without increased filling pressure^18,19^. In contrast, patients in this study developed a significant increase in LV diastolic pressures with dyspnea despite low workload and very modest rise in heart rate that led to exercise termination. The lack of increase in LV early diastolic filling rate reflects the opposing effects of LV relaxation and filing pressures on filling rate. In addition to abnormal diastolic function, cardiac contractile reserve appears compromised which along with the lack of an increment in peak filling rate precluded the increase in stroke volume with exercise. In this regard, in comparison to non-transplant patients with HFpEF who develop an increase in both wedge pressure and stroke volume with the exercise protocol used in this study^11^, the transplant population has two disadvantages: the abnormal diastolic function response to exercise and the lack of an increase in LV stroke volume. Interestingly, the exploratory analysis with biomarkers showed abnormal levels of interleukin-6 and TIMP-1 in several patients, suggesting they could serve as potential markers of the disease.

Heart transplantation is live saving in patients with end stage heart failure^20^. Despite transplantation, many patients have symptoms several years after surgery including exertional dyspnea and fatigue^21^. Some of these patients have reduced LV EF, but there is a paucity of data on their LV diastolic function and whether they have HFpEF. In fact, there are studies attributing the symptomatic status to anemia, chronic kidney disease, pulmonary pathology, and psychiatric reasons^22^. Aside from activation of inflammation and increased fibrosis, the incomplete sympathetic re-innervation many years after surgery can contribute to the absence of the normal shortening of τ with exercise^23^. Instead, lengthening of τ with exercise occurred in the majority of patients in this cohort.

To our knowledge, this is the first study to comprehensively evaluate LV systolic and diastolic properties in heart transplant recipients using the invasive gold standard of acquiring pressure volume loops with a conductance catheter to study cardiac function. LV stiffness was assessed by several analytic approaches and all methods led to consistent conclusions. Interestingly, the curve fitting constant (A) in this group of patients at 1.3 was very similar to that reported in non-transplant patients with HFpEF at 1.2^24^, which supports relying on the stiffness constant (β) cutoff value of 0.015^6^ to identify patients with increased chamber stiffness. In comparison with invasive measurements, echocardiographic indices of diastolic function did not detect the presence of abnormal LV relaxation or the increased LV chamber stiffness constant. This highlights the need for invasive data to diagnose the presence of diastolic dysfunction and HFpEF in several patients from this population.

Heart transplant recipients have several reasons that can lead to HFpEF. These include obesity, hypertension, diabetes, older age of donor heart, chronic allograft vasculopathy, and recurrent rejection episodes causing replacement and interstitial fibrosis. In addition, cytomegalovirus infection is associated with upregulation of MHC on endothelial cells with increased lipid content. The patients in this study had many of the above noted reasons for HFpEF which could serve as potential targets in a treatment regimen as well as in identifying high risk patients with intervention to be carried out before the development of HFpEF.

The study draws attention to the potential role of inflammation and fibrosis in the development of HFpEF in this patient population. CRP is a pentameric protein with an established role in systemic inflammation. Previous work showed worse graft survival in patients with CRP > 1.06 mg/L^25^. Of note, all patients in this study had a CRP level that exceeded the above threshold with a mean increase of 177.5%. In addition to CRP, IL-6 was abnormally elevated in the majority of patients. Likewise, GDF-15 level at 1.6 ng/mL in this series of heart transplant recipients is similar to levels reported in HFpEF patients where the median level was 1.08 ng/mL and exceeded the 0.6 ng/mL level in normal controls^26^. Further understanding of the mechanisms involved linking these two processes can result in effective therapy not only for longer graft survival but also for prevention of HFpEF.

Data were limited to 25 patients due to the invasive approach to generate pressure volume loops and the exclusion criteria, in addition to several patients declining to participate. There was some heterogeneity in the medications used in the study sample which could affect cardiac function. Nevertheless, the hemodynamic findings at baseline and with exercise were not different between patients who were on beta-blockers/calcium channel blockers and those who were not. Exercise testing during cardiac catheterization was not accompanied by measurements of peak oxygen consumption, and thus we could not link the abnormal LV systolic and diastolic function to exercise limitations. We did not evaluate for microvascular disease which is known to affect cardiac function in patients with HFpEF. Myocardial samples could have provided additional insights. However, patients received their transplants ≥ 5 years without concerns for rejection. We could not justify to IRB obtaining cardiac biopsies at time of cardiac catheterization. No control group was included as normal hemodynamic response to exercise is well established^11,13,18,19^. In that regard, patients with cardiac transplantation within 5 years would not be suitable controls since they can have myocardial abnormalities related to rejection per se, and our objective was to look at graft function in the absence of rejection at a stage when other factors that influence cardiac function play a role. Adding multivariable regression analysis could have provided additional insights into the main determinants of the hemodynamic findings. This could not be pursued due to the small sample size and collinearity. The wide range of time (6–21 years) between transplantation and inclusion in the study is another limitation.

While patients exercised to exhaustion, the increment in heart rate and blood pressure were modest. This may have accounted for the lack of an increase in measurements of LV systolic properties with exercise. Notwithstanding, the question of LV systolic function contribution to exercise limitations, LV diastolic function abnormalities were consistently demonstrated in this group of patients by several analytic methods using data acquired invasively.

This study draws attention to the diagnosis of HFpEF in heart transplant recipients. Many of the HFpEF patients in the study would not have been diagnosed without exercise assessment of LV diastolic pressures or invasive indices of LV diastolic function. Accordingly, it is reasonable to consider the invasive measurement of mean wedge pressure and cardiac output or LVEDP with exercise in symptomatic heart transplant recipients with normal LV filling pressures at rest. This could be achieved in a similar approach to what is used in patients with HFpEF using right heart catheterization for measurement of wedge pressure and cardiac output at rest and with graded exercise. Aside from trying to achieve a more satisfactory control of blood pressure, diuretics can be prescribed, and their doses escalated based on the cardiac catheterization results.

In addition to the above clinical implications, the findings add to the growing body of evidence linking inflammation and fibrosis to the abnormal cardiac structure and function in HFpEF in a clinical model where the immune system is activated.

## Data Availability

The datasets analyzed during the current study are available from the corresponding author on reasonable request.
